# The Geological Characteristics of the Vadose Zone Influence the Impact of Treated Wastewater on the Groundwater Quality (SCA.Re.S. Project 2019–2020)

**DOI:** 10.3390/pathogens11060677

**Published:** 2022-06-11

**Authors:** Osvalda De Giglio, Francesco Triggiano, Francesca Apollonio, Chrysovalentinos Pousis, Carla Calia, Giusy Diella, Francesco Bagordo, Sapia Murgolo, Tiziana Grassi, Cristina De Ceglie, Silvia Brigida, Giuseppina La Rosa, Pamela Mancini, Giusy Bonanno Ferraro, Antonella De Donno, Giuseppe Mascolo, Maria Clementina Caputo, Maria Teresa Montagna

**Affiliations:** 1 Department of Biomedical Science and Human Oncology, University of Bari Aldo Moro, Piazza G. Cesare 11, 70124 Bari, Italy; osvalda.degiglio@uniba.it (O.D.G.); francesco.triggiano@uniba.it (F.T.); francesca.apollonio@uniba.it (F.A.); chrysovalentinos.pousis@uniba.it (C.P.); carla.calia@uniba.it (C.C.); giusy.diella@uniba.it (G.D.); 2 Department of Pharmacy—Pharmaceutical Sciences, University of Bari Aldo Moro, Via E. Orabona 4, 70125 Bari, Italy; francesco.bagordo@uniba.it; 3 Consiglio Nazionale delle Ricerche (CNR), Istituto di Ricerca Sulle Acque (IRSA), Via F. De Blasio, 5, 70132 Bari, Italy; sapia.murgolo@ba.irsa.cnr.it (S.M.); cristina.deceglie@ba.irsa.cnr.it (C.D.C.); silvia.brigida@ba.irsa.cnr.it (S.B.); giuseppe.mascolo@ba.irsa.cnr.it (G.M.); maria.caputo@ba.irsa.cnr.it (M.C.C.); 4 Department of Biological and Environmental Science and Technology, University of Salento, Via Monteroni 165, 73100 Lecce, Italy; tiziana.grassi@unisalento.it (T.G.); antonella.dedonno@unisalento.it (A.D.D.); 5 Department of Environment and Health, Istituto Superiore di Sanità, 00161 Rome, Italy; giuseppina.larosa@iss.it (G.L.R.); pamela.mancini@iss.it (P.M.); giusy.bonannoferraro@iss.it (G.B.F.); 6 Consiglio Nazionale delle Ricerche (CNR)—Istituto di Ricerca per la Protezione Idrogeologica, Via Amendola 122 I, 70126 Bari, Italy

**Keywords:** wastewater treatment plant, groundwater, contaminants of emerging concern, chemicals, enteric viruses, porous aquifer, karst aquifer

## Abstract

This study evaluated whether some chemical and microbial contaminants in treated sewage effluents from two wastewater treatment plants (WWTP) reached the groundwater when they drained through a fractured karst vadose zone (WWTP-K) and a porous vadose zone (WWTP-P). Forty-five samples of sewage water (SW), treated water (TW), and monitoring well (MW), collected from WWTP-P (24) and WWTP-K (21), were analyzed for a range of microbiological and chemical properties. The *E. coli* and Salmonella counts were below the limits outlined in the Legislative Decree 152/06 in effluents from both types of WWTP. Enteric viruses were found in 37.5% and 12.5% of the SW and TW from WWTP-P, respectively. The percentages of Pepper mild mottle virus isolated were higher in TW (62.5% in WWTP-P, 85.7% in WWTP-K) than in SW and MW. The residual concentrations of contaminants of emerging concern (CEC) of each drug category were higher in the MW downstream of WWTP-K than of WWTP-P. Our results showed that the porous vadose zone was more effective at reducing the contaminant loads than the fractured karst one, especially the CEC, in the effluent. The legislation should include other parameters to minimize the risks from treated effluent that is discharged to soil.

## 1. Introduction

Worldwide, groundwater represents the main source of water for drinking purposes and agricultural and livestock needs [1]. In arid and semiarid areas, groundwater is used to meet the needs of the population where surface water resources are scarce or overexploited. In Europe, about 75% of the population depends on groundwater for their water supply [2]. There is concern that the groundwater quality has deteriorated, and its suitability for drinking or irrigation has been threatened, as groundwater overexploitation and anthropic pressures on aquifers from urbanization, industrialization, intensive agriculture, and animal husbandry have increased in recent decades [3,4,5,6,7]. The groundwater quality is affected by anthropic activities that impact on its chemical and microbiological parameters, and by the geological features of the rocks that host the groundwater through their role in determining the vulnerability of the aquifer to contamination [8,9,10].

Fractured and karst vadose zone makes aquifers more vulnerable to contamination than porous one, because they facilitate rapid transport of water through its fractures and/or karst conduits [8,11], meaning that there is less attenuation of contaminants in karst than in the porous vadose zone that act as a filter. When the water flows relatively slowly through the vadose zone, the pollutants can be removed by chemical reactions and other physical attenuation mechanisms [11]. Because of the characteristics of different aquifer types, an understanding of the processes that occur in the vadose zone is fundamental for preserving the groundwater quality [12,13]. Research is therefore needed on how contaminants move through the unsaturated zone before reaching the water table, particularly in close proximity to waste disposal and treatment facilities [14].

Municipal wastewater treatment plants (WWTPs) are full-scale bioreactors that are designed to treat domestic sewage from urban centers before it is released into the environment [15]. It is well known that WWTPs can remove most of the organic matter and microbiological contaminants in wastewater by conventional processes that are based on chemical and biological treatments, thereby reducing the risks to ecosystems and human health from effluents that are discharged into the environment [16]. Nevertheless, WWTP effluents discharged onto soil could be an important source of groundwater contamination, especially when WWTPs do not work adequately, such as when they are not large enough to cope with the volume of sewage to be treated, or when the wastewater systems malfunction, fail, or are unable to remove some categories of contaminants, for example, antibiotic-resistant bacteria [17,18]. Numerous researchers have documented the occurrence of trace compounds (mostly organic) in effluents, surface water, and drinking water, and have provided evidence that WWTPs do not efficiently remove several micropollutants from municipal wastewaters [19,20,21]. In Italy, the emission limits for urban and industrial wastewater discharges onto soil are outlined in Legislative Decree No. 152 of April 3, 2006 [22]. This legislation refers to numerous chemical parameters and one microbiological parameter, *Escherichia coli* (<5000 colony forming units, CFU/100 mL).

While guidelines have been published for some pollutants classified as ‘contaminants of emerging concern’ (CECs), there are no discharge limits. To support water policy and prioritization exercises, a list of organic compounds that must be monitored in water across the European Union was established in the EU Decision 2020/1161 on 4 August 2020 [23]. This list includes pharmaceuticals, personal care products, drugs, and steroid hormones that have been detected globally in the aquatic environment, and about which there is concern because of the possible effects on human health [24,25].

The Evaluation of Sanitary Risk Related to the Discharge of Wastewater to the Ground project (SCA.Re.S. project) was performed in the Salento Peninsula, Apulia, Southern Italy, in 2019–2020 in order to investigate the role of the vadose zone in retaining chemical and microbial contaminants from treated wastewater and the implication for the groundwater quality in different settings. In particular, two different hydrogeological domains, characterized by different lithological compositions of the vadose zones below two selected WWTPs, were examined: a karst fractured vadose zone (WWTP-K) and a porous vadose zone (WWTP-P), respectively.

Some of the results of this project, concerning changes in the groundwater quality recharged by the treated wastewater that passed through fractured and karst vadose zone, have already been reported [9]. Numerous researchers have studied the flow of contaminants through karst soil [9,10,11,26,27,28] but few have studied non-karst soil [29,30,31].

The aim of this paper therefore was (i) to report the results from monitoring contaminant transport through a porous vadose zone (WWTP-P), (ii) to compare the quality of the effluents from WWTP-K and WWTP-P before and after draining through karst and porous vadose zones, and (iii) to evaluate the role of the vadose zone on the groundwater quality in removing contaminants from WWTP effluent.

## 2. Results and Discussion

### 2.1. Chemical and Physical Parameters

The mean values (± standard deviations) of the chemical and physical parameters measured in three types of samples collected from the two WWTPs during the monitoring period are shown in Table 1.

All the values of biochemical oxygen demand (BOD_5_) and chemical oxygen demand (COD) for the treated wastewater (TW) were below the limits allowed by Lgs. D. 152/06. This leads us to affirm that, in general, the wastewater treatment process in both plants proved to be effective against organic substances. The parameters relating to the nutrients contained in TW showed that the total nitrogen (TN) was sufficiently reduced, while total phosphorus (TP) was slightly higher than the limit (2 mg/L) at both the treatment plants. 

Overall, the values of all parameters decreased significantly (*p* < 0.05), from sewage water (SW) to the monitoring wells (MW). However, it should be noted that the value for COD, an indicator of organic pollution, was always negative in the MW of WWTP-P, while the mean value was of 2.5 ± 3.1 mg/L in WWTP-K with a significant difference (*p* < 0.05) between the two plants.

### 2.2. Bacteria

The results from tests for the bacterial parameters in the WWTP effluents, expressed as mean concentrations (± SD), are presented in Table 2. The mean loads of *E. coli* were always below the limit allowed by Italian Lgs. D. 152/06 (5000 cfu/100 mL). *E. coli* and enterococci were detected in the effluents from both WWTP-P (893 ± 1138) and WWTP-K (6 ± 5) but were not detected in the respective MWs. 

*C. perfringens* was detected in TW and MW (in one of eight samples) from WWTP-P, and was detected in TW from WWTP-K. This microorganism results in being more resistant in the environment and to disinfection treatments than coliforms and enterococci [32]. It is the main etiological agent of myonecrosis of connective tissues [33] and may also be responsible for food poisoning and diarrhea [34].

*P. aeruginosa* was higher in the MW samples than in the TW samples at both the porous and karst sites. It is a free-living bacterium that mainly occurs in soil and natural waters (lakes, rivers, and groundwater), therefore the concentration of *P. aeruginosa* increased during the filtration process both in porous and karst fractured aquifer. It is one of the most important opportunistic pathogens of humans involved in a variety of infections, including respiratory and urinary tract infections, wound and soft tissue infections [35].

*Salmonella* spp. (results not shown) was not detected either at WWTP-K or WWTP-P.

The transport of bacterial cells through a porous soil is influenced by several mechanisms such as physical straining, which in turn depends on the pore size, the bacterial cell size, and the hydraulic load rate, as well as adsorption to porous soils, which, on the other hand, is affected by the content of organic matter, the development of biofilm and electrostatic attraction due to ion strength of the solution or electrostatic charges of cell- and particle surfaces [36,37]. Furthermore, the concentration of viable cells in groundwater may depend on the inactivation rate of microorganisms during the percolation phase which is influenced by abiotic and biotic factors such as moisture content, pH, temperature, organic matter, bacterial species, predation, and antagonistic symbiosis among microorganisms [38,39].

### 2.3. Enteric Viruses

The results from testing for viruses in the samples from both plants by nested PCR are shown in Table 3. In WWTP-P, enteric viruses were detected in 37.5% of SW samples and 12.5% of TW samples but were not detected in MW. NoV-GII was detected in SW (12.5%) and TW (12.5%), while NoV-GI (12.5%) and EV (12.5%) were only detected in SW. 

In WWTP-K, enteric viruses were found in 85.7% of SW samples, 57.1% of TW samples, and 14.3% of MW samples. Multiple virus families were detected in 85.7% of SW samples (up to three virus species in a single sample) and 28.5% of TW samples (up to two virus species in a single sample). NoV-GII and AdV were the most commonly detected viruses in SW (71.4%) and TW (42.9%). AdV was also found in MW (14.3%), while EV (57.1%) and NoV-GI (28.6%) were only detected in SW. 

HEV, HAV, and RoV were not detected in any samples. Pepper mild mottle virus (PMMoV) was isolated in all samples of both WWTPs except for MW of WWTP-P and was isolated at a higher percentage in TW (62.5% in WWTP-P, 85.7% in WWTP-K) than in the other samples. 

Six groups of enteric viruses were selected from the wide range of viruses excreted in human waste for this study because of their epidemiological significance as waterborne pathogens. Of these, only three, AdV, NoV, and EV, were detected in the analyzed water samples. It is important to note that molecular methods are only capable of detecting viral genomes, and do not provide information about infectivity, and thus positive samples do not necessarily indicate an actual threat to human health.

Viruses were present in the WWTP-P monitoring wells, perhaps because karst aquifers, unlike fissured and porous aquifers, are characterized by holes and channels through which water can flow rapidly, thereby transporting contaminants over great distances with little attenuation [27]. The degree of virus attachment is affected by several factors, including viral surface properties, groundwater quality, sediment surface charges, and soil moisture [40]. Further research will be carried out at the lab scale as part of the SCA.RE.S project to examine the processes of adsorption and/or inactivation of viruses through the unsaturated zone before reaching the groundwater. 

### 2.4. Contaminants of Emerging Concern

Using targeted screening (Section 3.6), 35 CECs and their degradation products were identified and quantified in the TW and MW samples. All the substances were grouped into pharmacological categories, namely antimicrobials, anticonvulsants, nonsteroidal anti-inflammatory drugs, beta-adrenoceptor blocking agents, UV filters, antipsychotic drugs, antihistaminic drugs, antidiabetic drugs, and X-ray contrast media. 

The average concentrations of the CECs detected in the TW and MW samples of the WWTPs are listed in Table 4, along with the residual concentrations (RC) of each contaminant, expressed as the percentage of the detected concentration in MW compared with its concentration in TW. The average of the sum of the substances grouped by pharmacological category (in bold) and the respective RCs are also listed.

**Figure 1:**
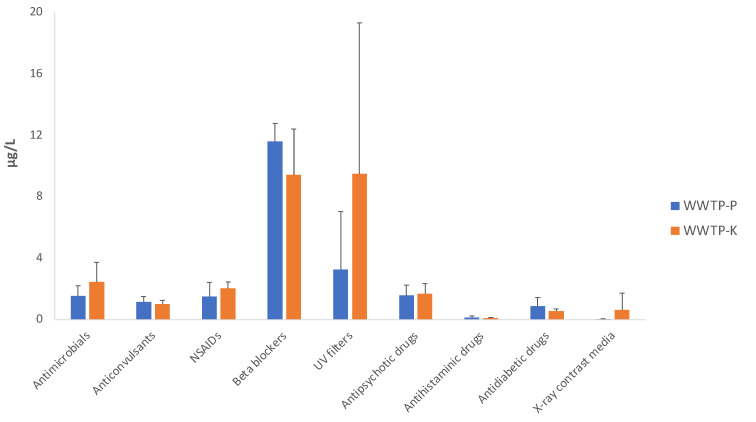
Concentrations of CECs grouped by pharmaceutical category in the WWTPs’ effluents (TW).

The concentrations of the detected CECs in the effluents of the two WWTPs did not differ significantly (*p* > 0.05) (Figure 1), perhaps because the two WWTPs use similar conventional processes to treat sewage. Moreover, studies have shown that the quantified contaminants are not efficiently removed during conventional wastewater treatment and are usually detected in secondary wastewater effluents at concentrations ranging from μg L^−1^ to ng L^−1^ [24].

Previous studies have reported that non-steroidal anti-inflammatory drugs (NSAIDs), b-blockers, antimicrobials, and anticonvulsants are widely used by the population and are widespread in WWTP influents [41,42]. Carbamazepine and lamotrigine were the main representatives of the anticonvulsant category, and they were detected at almost the same concentrations in the TW samples. Of the antimicrobials, fluconazole and levofloxacin were detected at the highest concentrations. 

Venlafaxine, a widely used antipsychotic, and fluconazole were included in the Third Watch List of the Water Directive Framework through the Decision (EU) 2020/1161. In the last EU Decision, there was no final conclusion about these substances, so it is important to monitor for their presence in the aquatic environment, and to have information to support a decision about their possible inclusion in the List of Priority Substances.

Olmesartan, irbesartan, and flecainide (b-blockers) were found at concentrations above 1 μg/L. In a recent study, flecainide was detected in almost all the effluents tested [43]. Ladhari et al. [44] found that, because of their low degradation rate, they were often released into the environment and detected in different water sources. 

Quantitatively, compounds in the beta-adrenoceptor blocking agent category, detected at average concentrations of 11.62 ± 1.14 μg/L in WWTP-P and 9.44 ± 2.98 μg/L in WWTP-K, were the most abundant in TW. Antimicrobials, with average concentrations of 1.53 ± 0.69 μg/L and 2.46 ± 1.27 μg/L in WWTP-P and WWTP-K, respectively, ranked second.

The average concentrations of 2-Phenyl-5-benzimidazolesulfonic acid (UV filter) were high in both WWTP-P and WWTP-K. The earlier study of WWTP-K showed that the concentrations of 2-Phenyl-5-benzimidazolesulfonic acid varied by season, as it was detected at different concentrations in summer and autumn.

The overall RC (%) results clearly highlighted that CECs behaved differently and had different fates in the porous and karst soils. The RCs of each pharmacological category were higher in the MW from WWTP-K than WWTP-P. These results probably reflect the hydrogeological characteristics of the areas where the plants are located.

WWPT-K is in a central-western area of Salento with karst fractured subsoil. This means that the groundwater is highly vulnerable, and contaminants can pass easily from the surface to the deep aquifer [45]. The groundwater velocity is usually high in karst aquifers that do not allow sufficient time for groundwater self-purification [11]. WWPT-P, on the other hand, is in the central-eastern part of the Salento Peninsula. The vadose zone is characterized by the presence of porous layers that could retain contaminants contained by the treated wastewater as it percolates through the subsoil. In the soil, CECs can undergo different processes, such as adsorption–desorption, transport, and biotic and/or abiotic transformations. The interactions between CECs and soil particles are also influenced by the properties of the soil (mainly the soil organic matter content, pH, clay content, and clay type) and the CEC molecular properties (such as lipophilicity, size, fractions of H-bond donors/acceptors, and charge) [42].

## 3. Materials and Methods

### 3.1. Study Scenarios

This project was carried out in two WWTPs of the Salento Peninsula (Southern part of Apulia Region, Italy) characterized by vadose zones of different compositions: one in an area mainly comprised of fractured and karst limestone and the other in an area mainly comprised of porous carbonate rock. 

The WWTPs located nearby the town of Carpignano Salentino (WWTP-P), and the town of Soleto-Galatina (WWTP-K) discharge effluent into infiltration pond systems from where it infiltrates through the vadose zone, thereby exploiting its natural capacity for filtering microbiological and chemical contaminants [46,47].

**Figure 2:**
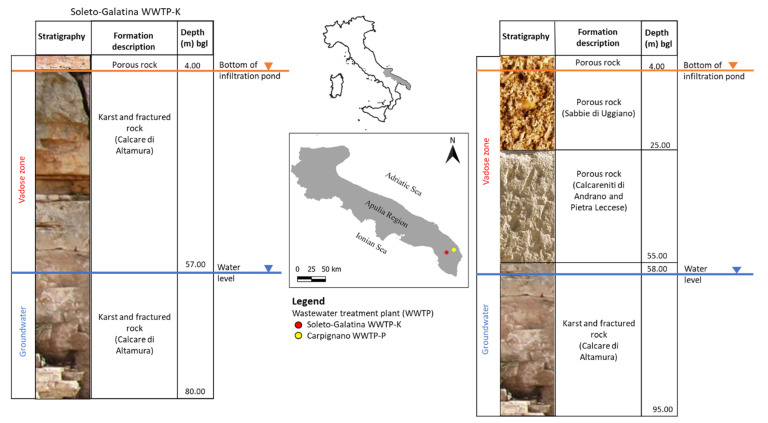
Schematic representation of the stratigraphy of the two studied wastewater treatment plants; WWTP-K = effluent flowing into a karst fractured vadose zone; WWTP-P = effluent flowing into a porous vadose zone.

The infiltration ponds of the Carpignano plant have been dug into the carbonate porous rock of the Pliocene, known as the Sabbie di Uggiano Formation. The vadose zone below the ponds, about 60 m thick, is constituted by a porous rock that belongs to the Calcareniti of Andrano and Pietra Leccese Formations, both of Miocene age. The groundwater lies in a layered aquifer system, characterized by a fault system-oriented NW-SE that separates into two distinct groundwaters: the shallow one hosted in the Calcarenite of Andrano formation, while the deeper one in the cretaceous fractured limestone belongs to the Calcare of Altamura formation, locally known as the Dolomie of Galatina formation, and stratigraphically located below the Pietra Leccese (Figure 2).

Instead, the bottom of the infiltration ponds of Soleto-Galatina plant corresponds to the top of the fractured and karstic limestone belonging to Calcare di Altamura Formation of Cretaceous age. Here, the vadose zone, about 57 m thick, is constituted by limestone, dolomitic limestone, and dolomite that are considerably diaclased, karstified, and often faulted. These rocks have a high degree of permeability. The groundwater lies in the deep carbonate aquifer and flows mainly in the fractures and within secondary karst conduits.

### 3.2. Sampling

**Figure 3:**
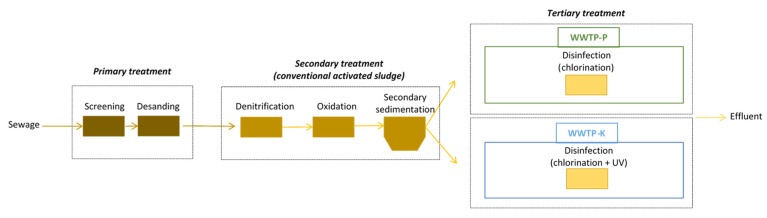
Schematic representation of the treatment processes of the two selected wastewater treatment plants; WWTP-K=effluent flowing into a karst fractured vadose zone; WWTP-P=effluent flowing into a porous vadose zone.

Both plants perform primary treatments, such as screening and sand separation, followed by secondary treatment such as denitrification, oxidation, and secondary sedimentation. Before being released onto the soil through a system of infiltration trenches, the effluents undergo a disinfection treatment (tertiary treatment), which is done by chlorination (sodium hypochlorite) in the WWTP-P, and by chlorination (sodium hypochlorite) and UV rays in the WWTP-K (Figure 3).

The quality of the wastewater before and after the secondary treatment and of the groundwater from wells close to the treatment plants was assessed from the chemical, physical, and microbiological properties. 

Samples were collected monthly from May 2019–December 2020. On the collection day, samples were collected between 8:00 and 11:00 in calm atmospheric conditions, with no rain.

A total of 45 water samples (24 from WWTP-P and 21 from WWTP-K) were collected, comprising sewage water (SW) (8 from WWTP-P and 7 from WWTP-K), treated water (TW) (8 from WWTP-P and 7 from WWTP-K), and water from a monitoring well (MW) (8 from WWTP-P and 7 from WWTP-K). The monitoring wells were located no more than 500 m downstream from each WWTP and were used routinely to monitor the plant activity by local authorities. 

Microbiological, chemical, and physical parameters, including some beyond those required by Italian law [22], were determined in the TW and MW samples. Samples of water for bacterial property analysis (2 L) were collected in sterile glass containers by using an automatic sampler. Samples of different volumes (2.5 L samples of SW, 40 L of TW, and 1000 L of MW) were also collected and analyzed for virus detection. Samples of TW and MW were collected and analyzed for chemical and physical properties (1 L) and CECs (200 mL) determination.

### 3.3. Detection of Bacterial Indicators

Bacterial indicators of fecal contamination (*Escherichia coli*, Enterococci, Salmonella spp., *Pseudomonas aeruginosa*, *Clostridium perfringens*) were assessed in the TW and MW water samples.

The UNI EN ISO 9308-1:2017 [48] method was used to detect the *E. coli*. The samples (100 mL) were filtered through cellulose ester membrane filters (47 mm Ø and 0.45 μm-pore size; Millipore Corporation, Bedford, MA, USA). The membranes were placed on plates containing Chromogenic Coliform Agar (Biolife Italiana Srl, Milan, Italy) and incubated at 36 ± 2 °C for 24 ± 2 h. 

The EN ISO 7899-2 (2003) method [49] was used to detect the Enterococci. The samples (100 mL) were filtered through cellulose ester membranes (47 mm Ø and 0.45-μm pore size; Millipore, Milan, Italy). The membranes were placed over a Slanetz and Bartley agar medium (Biolife Italiana srl, Milan, Italy) and incubated at 36 ± 1 °C for 48 h.

To detect *Salmonella* spp., a 1000 mL aliquot of each sample was filtered through a cellulose nitrate membrane filter (0.45-μm pore size) that was successively transferred into buffered peptone water (BPW) (Biolife Italiana srl, Milan, Italy) and incubated for 18–24 h at 36 ± 1 °C. A 0.1-mL aliquot of culture was inoculated into 10 mL of Rappaport Vassiliadis broth (Microbiol and C. s.n.c, Uta, Italy) and incubated at 41.5 °C for 24 + 24 h.

The broth was then streaked on xylose lysine deoxycholate agar plates (Biolife Italiana srl) and Hektoen Enteric Agar (Merck, Darmstadt, Germany). After incubating for 24 h at 36 ± 1 °C, the colonies with a typical morphology were sub-cultivated on Tryptic Soy Agar plates (Biolife Italiana srl) at 36 ± 1 °C and biochemically confirmed using the API 20E test (Biomèrieux, Marcy l’Etoile, France). Finally, colony typing was carried out using specific serological tests, as described in the APAT CNR IRSA manual 7080 [50].

To detect *P. aeruginosa*, 250 mL of each sample was filtered through a cellulose ester membrane filter (0.45-μm pore size). The membrane was placed onto a plate containing Pseudomonas selective agar supplemented with cetrimide (0.20 g) and nalidixic acid (15 mg) (Microbiol, Cagliari, Italy) and incubated at 36 ± 2 °C for 44 ± 4 h. Blue–green pyocyanin producing colonies were confirmed to be *P. aeruginosa* [51].

To detect *C. perfringens*, 100 ml of each sample was pre-treated at 75 ± 5 °C for 15 ± 1 min in a water bath and then filtered through a cellulose nitrate membrane filter (0.45-μm pore size). The membranes were placed on Tryptose Sulfite Cycloserine Agar (Biolife Italiana srl, Milan, Italy) and the plates were incubated at 44 °C for 24 h under anaerobic conditions (GasPak EZ Gas Generating Pouch Systems - BD Diagnostics, 7 Loveton Circle, Sparks, Maryland, USA). Black colonies were considered spores of sulfite-reducing clostridia [52].

### 3.4. Detection of Viruses

Molecular methods were used to test samples of SW, TW, and MW for different enteric viruses, namely adenovirus (AdV), norovirus genogroups I and II (NoV-I and NoV-II), enterovirus (EV), hepatitis A virus (HAV), hepatitis E virus (HEV), and rotavirus (RoV). The samples were also tested for the Pepper mild mottle virus (PMMoV).

Before molecular analysis, composite samples over a 24 h period were collected from the WWTP influent (SW) post the inlet screens (250 mL) and then concentrated using the two-phase (polyethylene glycol,PEG-dextran) separation method, in line with the method in the WHO guidelines for environmental surveillance of poliovirus circulation [53]. Samples from TW (40 L) and MW (1000 L) were filtrated by Nanoceram electropositive cartridges (Argonide Corporation, Sanford, FL, USA) and concentrated by the virus adsorption–elution (VIRADEL) technique, as described by Iaconelli et al., 2017 [54], and Montagna et al., 2020 [9]. 

Viral nucleic acid was extracted from concentrated samples after chloroform treatment (5 mL) using a semi-automated nucleic acid extraction platform (NucliSENS MiniMag, bioMerieux, Marcy l’Etoile, France), following the manufacturer’s instructions. Extracted RNA was stored at −80 °C until the molecular analysis.

Enteric viruses and PMMoV were detected by reverse transcription-nested polymerase chain reaction (PCR), following the method described by Bonanno Ferraro et al., 2021 [55]. 

The PCR products were loaded onto 2% agarose gel containing GelRed staining (Biotium, Fremont, CA, USA). The amplified products were purified using a Montage PCRm96 Microwell Filter Plate (Millipore, Billerica, MA, USA) and subjected to Sanger sequencing on both strands (Bio-Fab Research, Rome, Italy). Consensus sequences from each sample were compared with those available in the GenBank database using BLAST [56].

### 3.5. Chemical and Physical Parameters

The samples were analyzed for the physical and chemical parameters required by Lgs D. 152/06 [22]. The temperature, pH, and conductivity were measured in situ with a multiparameter probe (WTW MultiLine P4) with the potentiometric [57] and conductivity [58] methods. Total suspended solids (TSS) were determined by filtering samples through glass fiber filters [59], the biochemical oxygen demand (BOD5) was determined by the respirometric method [60], and the chemical oxygen demand (COD) was detected using the sealed tube method from the International Standard Organization [61]. The total nitrogen (TN) concentrations were measured by UV spectrometry after oxidative digestion with sodium persulfate using an actuator that operates in a coordinated analytical sequence [62]. The nitrate (NO_3_) concentrations were determined from the dissolved anions by liquid phase ion chromatography [63], and the TP concentrations were determined with the ammonium antimony-phospho-molybdate colorimetric method after sequential analysis [64].

### 3.6. Contaminants of Emerging Concern 

The TW samples from WWTP-P and the MW samples from the whole project period were examined for CECs. The detection was performed with a liquid chromate graphy system (Ultimate 3000; Thermo Fisher Scientific, Waltham, MA, USA) interfaced with a high-resolution mass spectrometer (TripleTOF® 5600+; AB Sciex, 500 Old Connecticut Path, Framingham, MA 01701, USA). The UPLC-QTOF/MS/MS conditions implemented for the detection and quantification of CECs followed a well-established method already described in the previous article [9]. 

Briefly, each sample was filtered through a 0.20 μm regenerated cellulose filter and spiked with an internal standard, i.e., CBZ D10, at a level of 10 ng/mL, and then was injected (2000 μL) using an optimized online solid phase extraction method (online SPE) that consisted of a pre-concentration column and an analytical column for analyte separation. 

The data were processed with AB Sciex software. Each sample was screened for the presence of target compounds by comparing the retention time and the MS/MS fragmentation pattern of the detected compounds with those of the standard compounds. A reference calibration curve was inserted into the 0.01–5 μg/L range (0.01, 0.05, 0.1, 0.5, 1, and 5 μg/L) to quantify all the compounds identified in the samples. 

### 3.7. Statistical Analysis

The results from the chemical and microbiological analysis were entered into a Microsoft Excel database and statistically processed using MedCalc Software version 12.3 (MedCalc Software bvba, Ostend, Belgium). The values of the microbiological parameters that were not detected were set at half (0.5 CFU/100 mL) of the detection limit (<1 CFU/100 mL), as described in Lorimer and Kiermer, 2007 [65].

The arithmetic mean, standard deviation, maximum, and the median were calculated for each group of quantitative variables. The D’Agostino–Pearson normality test was used to determine if the data sets conformed to a normal distribution, and the Tukey test was used to detect outliers. The RC of each contaminant of emerging concern in the MW was calculated as a percentage of the concentration of the same contaminant found in the TW. 

The Student’s *t*-test was used to determine if there were significant differences between the RCs of the substances grouped by pharmacological category at the different monitoring points.

## 4. Conclusions

The findings from the present investigation, combined with the findings from the earlier study, confirmed that, based on the chemical, physical, and microbiological measurements, the quality of the WWTP effluent discharged into the subsoil met the requirements of the current Italian Legislation. However, microbiological parameters beyond those required by the legislation, including viruses, were detected. The results demonstrate that parameters beyond *Salmonella* and *E. coli* should be included in the legislation that governs the discharge of effluent onto soil or into subsoil.

Of the karst subsoil and the porous soil, the porous one was better at retaining the chemical and microbial contaminants contained in the effluent from the WWTP-P, including emerging contaminants, probably because of its self-filtering capacity. This meant that effluent that had passed through the porous vadose zone was less likely to contaminate the groundwater than effluent that passed through the fractured and karst one. Thus, the risk of groundwater contamination is greater under a karst fractured vadose zone than under a porous vadose zone. In addition, the results confirm that pollutants are transported and diffuse more rapidly through a karst fractured domain than through a non-karst zone, without sufficient time to be retarded by chemical reactions or other attenuation mechanisms.

To date, few studies have considered how factors such as the precipitation rate, moisture, and seasonality influence the transport of effluents containing microbes through a non-karst vadose zone to the groundwater. A sound understanding of these processes would be beneficial for protecting the groundwater to ensure it was suitable for irrigation and drinking purposes. Therefore, further lab- and field-scale studies will be carried out in the SCA.RE.S project to investigate (1) the viability of enteric viruses in the WWTP effluents and (2) the variability of the groundwater quality recharged by effluent passing through vadose zones of different depths and lithology.

## Figures and Tables

**Table 1:** Chemical and physical parameters, reported as mean values (± standard deviation), measured in the sewage water (SW), treated water (TW), and monitoring well (MW) samples in the porous aquifer (WWTP-P), and karst fractured aquifer (WWTP-K).

Parameters	Unit of Measure	Limit Value	WWTP-P			WWTP-K		
		TW	SW	TW	MW	SW	TW	MW
pH		6–8	7.7 ± 0.3	7.8 ± 0.2	7.4 ± 0.1	7.7 ± 0.3	7.4 ± 0.3	7.4 ± 0.3
Conductivity	(μS/cm)	-	1745 ± 264	1318 ± 319	568 ± 36	1699 ± 210	1080 ± 265	1163 ± 35
BOD_5_	(mg/L)	20	640 ± 207	11.3 ± 4.6	0	580 ± 142	10.3 ± 2.5	0
COD	(mg/L)	100	935 ± 267	42.9 ± 45.5	0	859 ± 215	27.5 ± 6.5	2.5 ± 3.1
TN	(mg/L)	15	106.4 ± 16.7	14.0 ± 17.3	27.1 ± 2.2 *	74.1 ± 11.6	14.4 ± 9.2	26.4 ± 2.3 *
TP	(mg/L)	2	12.3 ± 4.0	3.4 ± 3.7	ND	10.7 ± 3.6	2.1 ± 0.7	ND

BOD_5_: biochemical oxygen demand, COD: chemical oxygen demand, TN: total nitrogen, * as nitrates (NO_3_^−^), TP: total phosphorus, ND: not detected.

**Table 2:** Average concentrations (±SD) of bacterial parameters in the treated water (TW) and monitoring well (MW) samples of the porous (WWTP-P) and the karst fractured (WWTP-K) aquifers.

Bacteria	Unit of Measure	WWTP-P		WWTP-K	
		TW	MW	TW	MW
*E. coli*	CFU/100 mL (P/T)	893 ± 1138 (8/8)	<1 (0/8)	6 ± 5 (7/7)	<1 (0/7)
Enterococci	CFU/100 mL (P/T)	16.1 ± 30 (8/8)	<1 (0/8)	5 ± 11 (7/7)	<1 (0/7)
*C. perfringens*	CFU/100 mL (P/T)	3 ± 3 (8/8)	2 ± 5 (1/8)	5 ± 2 (7/7)	<1 (0/7)
*P. aeruginosa*	CFU/250 mL (P/T)	3 ± 6 (8/8)	463 ± 1228 (8/8)	34.0 ± 89 (7/7)	5750 ± 7599 (7/7)

(P/T) = number of positive samples/number of analyzed samples.

**Table 3:** Percentage of virus positive samples in the sewage water (SW), treated wastewater (TW), and monitoring well (MW) samples from the porous (WWTP-P) and the karst-fractured (WWTP-K) aquifers, determined by PCR assay.

	WWTP-P			WWTP-K		
Virus	SW N (%)	TW N (%)	MW N (%)	SW N (%)	TW N (%)	MW N (%)
AdV	0 (0)	0 (0)	0 (0)	5 (71.4)	3 (42.9)	1 (14.3)
NoV-GI	1 (12.5)	0 (0)	0 (0)	2 (28.6)	0 (0)	0 (0)
NoV-GII	1 (12.5)	1 (12.5)	0 (0)	5 (71.4)	3 (42.9)	0 (0)
EV	1 (12.5)	0 (0)	0 (0)	4 (57.1)	0 (0)	0 (0)
HEV	0 (0)	0 (0)	0 (0)	0 (0)	0 (0)	0 (0)
HAV	0 (0)	0 (0)	0 (0)	0 (0)	0 (0)	0 (0)
RoV	0 (0)	0 (0)	0 (0)	0 (0)	0 (0)	0 (0)
PMMoV	4 (50)	5 (62.5)	0 (0)	5 (71.4)	6 (85.7)	3 (42.9)

AdV: adenovirus, NoV: norovirus; EV: enterovirus; HAV: hepatitis A virus; HEV: hepatitis E virus; RoV: rotavirus; PMMoV: pepper mild mottle virus.

**Table 4:** Mean CEC concentrations (± standard deviation) and the related pharmacological categories (in bold) in the treated wastewater (TW) and monitoring well (MW) samples of the porous (WWTP-P) and the karst fractured (WWTP-K) aquifers.

Name	WWTP-P			WWTP-K		
	TW (μg/L)	MW (μg/L)	RC (%)	TW (μg/L)	MW (μg/L)	RC (%)
**Antimicrobials**	**1.53 ± 0.69**	**0.01 ± 0.01**	**1.41 ± 2.43**	**2.46 ± 1.27**	**0.49 ± 0.11**	**41.29 ± 52.73**
Clarithromycin	0.04 ± 0.05	0.00 ± 0.01	3.33 ± 8.16	0.10 ± 0.05	0.00 ± 0.01	1.66 ± 4.39
Climbazol	0.02 ± 0.03	0.00	0.00 ± 0.00	0.08 ± 0.03	0.04 ± 0.05	52.25 ± 36.76
Fluconazole	0.62 ± 0.28	0.01 ± 0.01	2.12 ± 2.54	0.38 ± 0.14	0.43 ± 0.11	122.16 ± 33.04
Levofloxacin	0.84 ± 0.60	0.00	0.00 ± 0.00	1.89 ± 1.19	0.01 ± 0.02	0.89 ± 1.87
**Anticonvulsants**	**1.14 ± 0.35**	**0.02 ± 0.01**	**1.41 ± 1.22**	**1.02 ± 0.25**	**0.86 ± 0.12**	**78.53 ± 67.88**
Carbamazepine	0.39 ± 0.08	0.01 ± 0.01	2.34 ± 2.66	0.32 ± 0.07	0.49 ± 0.08	162.19 ± 55.75
Carbamazepine-10,11- Epoxide	0.11 ± 0.10	0.00 ± 0.01	0.31 ± 0.89	0.07 ± 0.02	0.04 ± 0.01	59.32 ± 25.46
Gabapentin	0.23 ± 0.18	0.00	0.00 ± 0.00	0.25 ± 0.10	0.00 ± 0.01	0.64 ± 1.70
Lamotrigine	0.41 ± 0.19	0.01 ± 0.01	2.42 ± 1.90	0.38 ± 0.14	0.33 ± 0.09	91.97 ± 33.01
**Nonsteroidal anti-inflammatory drugs**	**1.49 ± 0.92**	**0.001 ± 0.002**	**0.12 ± 0.35**	**2.04 ± 0.43**	**0.11 ± 0.05**	**19.60 ± 43.52**
Diclofenac	1.20 ± 0.71	0.00	0.00 ± 0.00	1.52 ± 0.42	0.00	0.00 ± 0.00
Ketoprofen	0.07 ± 0.13	0.00	0.00 ± 0.00	0.19 ± 0.08	0.00	0.00 ± 0.00
Niflumic Acid	0.04 ± 0.03	0.00	0.00 ± 0.00	0.07 ± 0.03	0.00 ± 0.01	1.19 ± 3.15
Tramadol	0.18 ± 0.09	0.00 ± 0.01	0.37 ± 1.04	0.25 ± 0.20	0.11 ± 0.05	77.23 ± 57.85
**Beta-adrenoceptor blocking agents**	**11.62 ± 1.14**	**0.01 ± 0.01**	**0.05 ± 0.05**	**9.44 ± 2.98**	**1.87 ± 0.54**	**9.30 ± 14.21**
Atenolol	0.02 ± 0.03	0.00	0.00 ± 0.00	0.08 ± 0.03	0.00 ± 0.01	4.80 ± 9.08
Bisoprolol	0.07 ± 0.07	0.00	0.00 ± 0.00	0.15 ± 0.07	0.01 ± 0.01	4.20 ± 5.40
Clopidrogel	0.02 ± 0.01	0.00	0.00 ± 0.00	0.02 ± 0.00	0.00	0.00 ± 0.00
Fenofibric Acid	0.04 ± 0.10	0.00	0.00 ± 0.00	0.09 ± 0.08	0.00	0.00 ± 0.00
Flecainide	1.76 ± 0.88	0.00 ± 0.01	0.17 ± 0.47	0.99 ± 0.22	0.07 ± 0.03	6.84 ± 3.05
Irbesartan	3.36 ± 0.76	0.00	0.01 ± 0.02	1.98 ± 0.75	0.14 ± 0.10	6.65 ± 4.37
Irbesartan 446	0.67 ± 0.16	0.00 ± 0.01	0.19 ± 0.35	0.89 ± 0.56	0.24 ± 0.10	32.36 ± 16.48
Losartan	0.06 ± 0.07	0.00	0.00 ± 0.00	0.05 ± 0.03	0.00	0.00 ± 0.00
Metoprolol	0.08 ± 0.05	0.00	0.00 ± 0.00	0.08 ± 0.04	0.01 ± 0.01	17.68 ± 11.79
Metoprolol Acid	0.18 ± 0.14	0.00	0.00 ± 0.00	0.45 ± 0.23	0.03 ± 0.02	7.76 ± 5.07
Olmesartan	3.75 ± 0.74	0.01 ± 0.01	0.31 ± 0.23	3.73 ± 1.35	1.36 ± 0.35	39.57 ± 11.69
Sotalol	0.19 ± 0.13	0.00	0.00 ± 0.00	0.14 ± 0.04	0.01 ± 0.02	7.64 ± 14.18
Telmisartan	1.11 ± 0.44	0.00	0.00 ± 0.00	0.25 ± 0.15	0.00	0.00 ± 0.00
Valsartan	0.31 ± 0.51	0.00	0.00 ± 0.00	0.55 ± 0.58	0.00	0.00 ± 0.00
**UV filters**						
2-Phenyl-5-BenzimidazolesulfonicAcid	3.28 ± 3.77	0.00	0.00 ± 0.00	9.50 ± 9.82	0.13 ± 0.23	2.74 ± 4.45
**Antipsychotic drugs**	**1.57 ± 0.66**	**0.001 ± 0.002**	**0.04 ± 0.11**	**1.67 ± 0.68**	**0.60 ± 0.19**	**38.58 ± 34.41**
Amisulpride	0.14 ± 0.07	0.00	0.00 ± 0.00	0.33 ± 0.19	0.12 ± 0.05	46.44 ± 26.93
EDDP	0.83 ± 0.38	0.00	0.00 ± 0.00	0.86 ± 0.42	0.25 ± 0.14	31.37 ± 14.04
Metamphetamine	0.00	0.00	-	0.07 ± 0.04	0.00	0.00 ± 0.00
Sulpride	0.27 ± 0.14	0.001 ± 0.010	0.15 ± 0.42	0.21 ± 0.07	0.14 ± 0.04	73.72 ± 38.02
Venlafaxine	0.31 ± 0.17	0.00	0.00 ± 0.00	0.19 ± 0.09	0.09 ± 0.03	52.37 ± 24.57
**Antihistaminic drugs**						
Cetirizine	0.13 ± 0.12	0.00	0.00 ± 0.00	0.10 ± 0.04	0.02 ± 0.02	22.35 ± 13.14
**Antidiabetic drugs**						
Sitagliptin	0.87 ± 0.55	0.00	0.00 ± 0.00	0.57 ± 0.12	0.11 ± 0.06	18.69 ± 8.88
**X-ray contrast media**						
Iopromide	0.02 ± 0.04	0.00	0.00 ± 0.00	0.62 ± 1.11	0.00 ± 0.01	0.05 ± 0.09

## Data Availability

All data generated or analyzed during this study are included in this published article.
